# Effect of Neutral and Acidic Protease Processing Intervals on Optimising Nutritional Value and Enhancing Physico-Chemical Properties of Oat Drink

**DOI:** 10.3390/foods13142285

**Published:** 2024-07-20

**Authors:** Monica Nabil Gayed Ibrahim, Helena Andreson, Sana Ben-Othman, Ivi Jõudu

**Affiliations:** Chair of Food Science and Technology, Institute of Veterinary Medicine and Animal Sciences, Estonian University of Life Sciences, Kreutzwaldi 56/5, 51006 Tartu, Estonia; helena.andreson@emu.ee (H.A.); sana.othman@emu.ee (S.B.-O.); ivi.joudu@emu.ee (I.J.)

**Keywords:** protease treatment, water-soluble proteins, β-glucan, oat drinks, stability, sensory attributes

## Abstract

This study aimed to maximise the content of water-soluble protein (WSP) and β-glucan (BG) in oat drink (OD) products by optimising the duration of treatment with neutral (NP) and acidic (AP) proteases. Additionally, it investigated the correlation between changes in the OD’s nutritional profile and its rheological and sensory properties. After initial treatment with α-amylase, the OD samples were divided into two groups, i.e., one treated with NP and the other with AP for 30, 60, 120, and 180 min. The samples were then analysed for their WSP and BG contents. Samples with an optimised treatment duration were evaluated for their rheological and sensory properties. The OD sample treated with AP for 60 min exhibited the highest β-glucan (0.52 g/100 mL) and WSP (1.56 g/100 mL) contents, improved storage stability, and the lowest sedimentation rate (2.13%/h), compared to the control OD sample. However, sensorially, this sample was characterised by a sticky, gluey mouthfeel and was less acceptable as a drinkable product. This study demonstrated the potential effect of protease treatment on enhancing the nutritional value and stability of OD products, although further studies are necessary to improve the sensory properties of these drinks.

## 1. Introduction

Over the past decade, major consumer research has emphasised the need to develop novel, nutritious, healthy, and functional plant-based dairy alternative beverages. The dominant factors driving current market demands include the prevalence of lactose intolerance, milk protein allergy, hypercholesterolemia, and obesity, alongside ethical and environmental concerns [1,2]. According to currently available products on the market, a variety of plant-based food materials, such as cereals, legumes, seeds, nuts, and pseudocereals, have been used to produce various plant-based beverages [1,3]. Among these, oat drink (OD) has become one of the typical representatives of newer and faster-developing cereal-based dairy alternatives in recent years due to its delicate and sweet aftertaste, nutritional benefits, and low environmental impact [2].

Due to the health benefits of oat grains, they have been recognised as a promising raw material for the production of oat-based functional beverages [4]. Oats are characterised by a balanced nutrient composition, being a good source of soluble fibre, particularly β-glucan, functional proteins, fats (especially unsaturated fatty acids), and phytochemicals [1,5]. The protein content in oats ranges from 11 to 15%, with the main protein being globulin, which is characterised by its low water solubility. Oats have been recognised as a source of high-quality protein with a good amino acid balance [6,7]. Compared to other grains, oats are notably rich in lysine while containing lower levels of glutamic acid and prolamin [6,8].

The interest in oat grains is driven by the presence of β-glucan, a functional active component with nutraceutical properties [2]. The β-glucan content in oat grains is estimated to be around 3.9–7.5 g/100 g of dry matter [4,5]. As a dietary fibre, oat β-glucan resists digestion and absorption in the small intestine, thereby attenuating both blood cholesterol and glucose levels, serving as a substrate for the gut microbiota, and promoting laxation [1,9]. The American Food and Drug Administration (FDA) and the European Food Safety Authority (EFSA) have approved a recommended daily dose of no less than 3 g of oat β-glucan, with a recommendation of 0.75 g per serving, to lower the risk of coronary heart diseases [10,11,12].

The production process of OD comprises multiple stages, beginning with the selection of raw materials, grinding, enzymatic hydrolysis, and filtration/decantation, followed by formulation, homogenisation, UHT treatment, and aseptic packaging [3,13]. The most crucial step in this production is the enzymatic treatment, where identifying the types of enzymes and their dosage influences the nutritional parameters and the acceptability of the product. Oat starch has a low gelatinization temperature ranging from 44.7 to 73.7 °C, which presents challenges in terms of yield, total solids, viscosity, and acceptability of oat drinks (ODs) [4]. Consequently, α-amylase utilisation has become a necessary treatment for hydrolysing oat starch into maltodextrins and glucose, thereby preventing starch gelatinization during heat treatment and facilitating the filtration process while also increasing the production yield of oat beverages [4,5].

Oat proteins, especially globulin, mainly exist in colloidal and unfolded, randomly coiled structures in aggregated form and have poor water solubility and emulsifying activity [7,14]. During the heat treatment of ODs, these proteins are easily denatured and aggregated, resulting in the formation of precipitates. Therefore, proteases such as papain, Flavourzyme (a mixture of refined *Aspergillus oryzae* proteolytic enzymes, including both endo- and exo-peptidases), and trypsin are introduced using a controlled/limited method to partially hydrolyse the protein into smaller molecules (polypeptides and amino acids), which results in the disappearance of the aggregation phenomenon in the OD, thereby improving its solubility and stability [7,8,15]. Triantafyllou [16] studied a second enzymatic category, i.e., ‘protein–glutaminase’, which specifically catalyses the deamidation of the side chain amino group of protein–bound glutaminyl residues, thereby improving protein solubility in oat products.

The utilisation of proteolytic enzymes in manufacturing ODs requires a detailed study of each protease type separately in order to select the optimal treatment time and ensure the preferred degree of hydrolysis for the protein. Moreover, previous research has not discussed the possible indirect effect of protease utilisation on β-glucan content in ODs, which may result from the firm covalent bond between the protein and the β-glucan in the oat structure, thereby creating a strong protein–glucan complex structure [17,18]. Because of the potential nutritional changes that can occur in the protease-treated OD, further research is needed to highlight their impact on altering the rheological and sensory properties of the product. Therefore, this study aimed to investigate the optimal duration of neutral and acidic protease treatments in OD processing to improve protein content and solubility. In addition, it examined the influence of each protease treatment on altering the β-glucan content and the physicochemical, rheological, and sensory properties of the final product. Moreover, this study investigated the relationship between the variation in the chemical composition of OD and the changes in its physicochemical and rheological properties.

## 2. Materials and Methods

### 2.1. Raw Materials and Enzymes

Dehulled oat grains produced by Tartu Mill AS (Tartu, Tartumaa, Estonia) and processed by Dobeles dzirnavnieks (Dobele, Dobeles novads, Latvia) were used for the preparation of the oat drinks. The nutritional composition of the oat grains was determined using the AOAC methods [19] at the Feed and Metabolism Research Laboratory and the Laboratory of Food Science and Technology at the Estonian University of Life Sciences. The results were as follows: 16.7% crude protein, 7.2% crude fat, 51.8% starch, 1.39% sugar, 2.5% crude ash, 1.3% crude fibre, and 4.2% β-glucan on a dry matter basis.

Pure α-amylase from *Bacillus licheniformis* (EC: 3.2.1.1, 3000 U/mL, Megazyme International Ltd., Bray, Co. Wicklow, Ireland), free of any other enzymatic residuals, including β-glucanase and proteases, was used for starch hydrolysis. The optimal conditions for its enzymatic activity were a temperature of 75 °C and a pH of 6.0–6.5. EnerZyme P7 (EC: 3.4.24.28), containing neutral protease (NP) from *Bacillus subtilis* (100 U/mL) with an optimum temperature of 45–55 °C and pH 5.7–7.0, and Distizym Protacid (EC: 3.4.23.18), containing acidic protease (AP) from *Aspergillus niger* (520 U/mL) with an optimum temperature of 50–58 °C and pH 3.0–5.0 (activity extends from pH 1.5–6.5), were used for protein hydrolysis. Both proteases were free from other enzyme traces and were purchased from Erbslöh Geisenheim GmbH (Geisenheim, Germany).

### 2.2. Experimental Design for Oat Drink Processing

A preprocessing roasting stage at 95 °C for 15 min was required for the dehulled oat grains to inactivate their endogenous enzymes without severely affecting their nutritional components [20]. This was followed by grinding in a knife mill (GM 300, Retsch GmbH, Haan, Germany) for 2.5 min at 3000 rpm and sieving for 5 min through graduated screen sieves (AS 200-digit cA, Retsch GmbH, Haan, Germany). Oat flour with a particle size of ≤1 mm was selected for the experiments.

**Figure 1:**
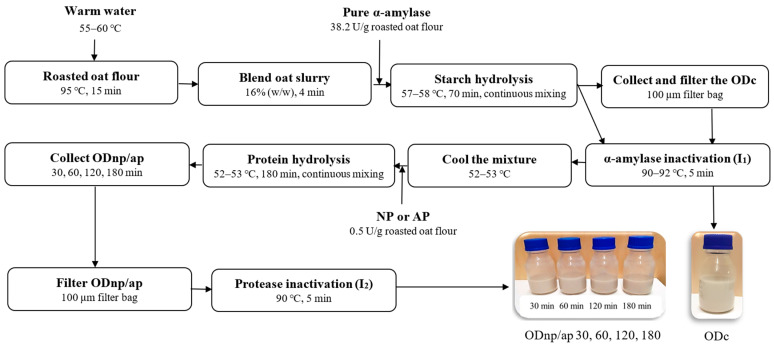
Schematic representation of the amylase and protease treatment in oat drink (OD) processing, where ODc is the control and ODnp/ap 30, 60, 120, and 180 are the oat drinks treated with either neutral protease (NP) or acidic protease (AP) for 30, 60, 120, and 180 min, respectively.

The OD processing was carried out using the technological processes presented in Figure 1. Oat slurry was obtained by blending roasted oat flour with warm water at a ratio of 1:5.25 (*w*/*w*) for 4 min using a Kenwood food processor 1500 W set at speed 4 (Kenwood Chef Titanium KMC070, Kenwood Chef Service limited, Bridgwater, Somerset, UK). The blended oat slurry was then treated with pure α-amylase at a dose of 38.2 U/g oat flour for 70 min, maintaining a temperature of 57–58 °C and a pH of 6.61 with continuous mixing using a magnetic stirrer, as described previously by Deswal et al. and Ibrahim et al. [4,5]. A control sample (OD_C_) was taken, filtered through a 100 µm filter bag, and inactivated (I_1_) by heating at 90–92 °C for 5 min.

The remainder of the slurry was directly inactivated (I_1_) and cooled to 52–53 °C, where its pH was determined to be 6.1–6.3. It was then divided into two halves; one half was treated with NP, and the other half with AP, each at a dose of 0.5 U/g oat flour. After treatment durations of 30, 60, 120, and 180 min, samples from each batch were collected, filtered through a 100 µm filter bag, and subsequently inactivated (I_2_) by heating at 90 °C for 5 min. For further analyses, all oat drinks (ODs) were stored at 4 °C for a week, except for the visual stability during the cold storage; the ODs were stored for a month.

### 2.3. Analyses Overview

The nutritional composition—total protein (TP), water-soluble protein (WSP), β-glucan (BG), and free amino nitrogen (FAN)—along with total solids content (T.S.) and production yield (PY), were analysed for the ODc, and all OD samples were treated with NP or AP for 30, 60, 120, and 180 min after stirring and preparation according to the procedure of each analysis. Following these analyses, the optimal duration for each protease treatment was selected based on the maximisation of the OD’s total protein, water-soluble protein, and β-glucan content. The selected OD samples from the NP and AP treatments were further evaluated for changes in their physicochemical–rheological properties (viscosity, visual cold storage stability, separation rate, density, Brix degree, and colour intensity) and sensory parameters compared to the ODc.

### 2.4. Nutritional Composition Analysis of Oat Drinks

The total protein content of the ODs was successfully measured using the Lowry method [21], leveraging the solubility of oat globulin (the major oat protein) in the weak alkaline conditions required for the Lowry reaction [22,23]. Prior to the analysis, 1 mL from each OD sample was diluted to 100 mL with distilled water and thoroughly mixed on a vortex. The absorbance of the samples was measured at a wavelength of 750 nm using a Specord 250 plus spectrophotometer (Analytik Jena GmbH+Co., Jena, Germany). A bovine serum albumin (BSA) standard curve was used to calculate the TP concentration (mg/L) in the diluted samples, which was then recalculated to express the TP content in g/100 mL. For WSP analysis, the diluted samples were centrifuged at 2295× *g* for 10 min using a Sigma 3–18KS universal refrigerated tabletop centrifuge (Sigma Laborzentrifugen GmbH, Osterode am Harz, Germany). The absorbance of the supernatants was then measured for WSP quantification in mg/L using the BSA standard curve, and the results were recalculated to express the WSP content in g/100 mL. 

The free amino nitrogen analysis was conducted with the EBC-ninhydrin colorimetric method as described by Lie [24]. Prior to analysis, OD samples were diluted 100-fold with distilled water, thoroughly mixed on a vortex, and then centrifuged at 2295× *g* for 10 min using a Sigma 3–18KS universal refrigerated tabletop centrifuge (Sigma Laborzentrifugen GmbH, Osterode am Harz, Germany). Absorbance was measured at a wavelength of 570 nm with a Specord 250 plus spectrophotometer (Analytik Jena GmbH+Co., Jena, Germany). A glycine standard solution was prepared, and its absorbance was recorded at the same wavelength to be used in the equation for FAN calculation (mg/L). The FAN content in the OD samples was subsequently expressed in mg/100 mL. 

The method for determining the β-glucan content involves a mixed-linkage β-glucan enzymatic assay kit (AACC method 32–23.01 and McCleary’s method, Megazyme International Ltd., Bray, Co., Wicklow, Ireland), as reported by Önning et al. [25]. The OD samples were prepared through two stages of ethanol precipitation at 70% and 50%, followed by a two-step hydrolysis with lichenase and β-glucosidase. The resulting glucose was detected using a glucose determination reagent containing glucose oxidase, peroxidase, and 4-aminoantipyrine. This reagent reacts with glucose to produce 4-N-(p-benzoquinoneimine)-antipyrine, which is detectable at 510 nm on a UV–vis spectrophotometer (Specord 250 plus, Analytik Jena GmbH+Co., Jena, Germany). The absorbance of the samples was compared to that of a standard 100 μg glucose solution; the difference in absorbance was then used to calculate the BG content [9,26]. 

### 2.5. Assessment of Oat Drink’s Physicochemical and Rheological Parameters

The total solids were estimated using AOAC Method 990.20 for the forced air oven-drying methodology [27]. The PY of OD was calculated as the percentage of the filtrate obtained from the hydrolysed oat slurry after the filtration process [4]. All subsequent physico-rheological measurements were carried out at room temperature (20–25 °C).

The viscosity of OD was determined using a controlled Brookfield DV-III ultra-programmable rheometer (Brookfield Engineering Laboratories, Inc., Middleboro, MA, USA) equipped with a spindle LV-1 at a rotation speed of 230 rpm for 120 s at 20 °C, as described previously by Ibrahim et al. [5]. The recorded average torques were 82.52% in ODc, 84.02% in ODnp, and 87.5% in ODap. The measurements indicated the OD viscosity (cP) at specific times. 

The separation rate (Sep. rate) was measured using an analytical centrifuge (Sigma 3–18KS, Sigma Laborzentrifugen GmbH, Osterode am Harz, Germany) with a rotational speed of 1721× *g* for 60 min at 24 °C [13]; the Sep. rate was expressed as %/h. 

The ODs were visually observed after 17 days of storage at 4 °C for product stability and phase separation assessment [5] since at this storage period, a clear difference between the phase separation of each sample appeared; moreover, some organoleptic changes appeared after this storage period, which could be the result of microbiological changes and affect the drink stability. A comparative photo of the three OD bottles (ODc, ODnp30, ODap60) was taken on the specified day using a Samsung Galaxy A52s 5G OIS camera (Samsung, Seoul, Republic of Korea) and processed using Paint.Net 5.0.8 version 2023 (dotPDN LLC, Washington, DC, USA) into 1380/843 pixels (width/height) and 8-bit sRGB. The separation tendency was determined by measuring the height of the separation phase formed at a specific storage period using a graduated bottle and expressed as a percentage of the total liquid height [9].

The product density (ρ) was determined by dividing the mass of the OD sample (approximately 31.5 g) by its volume in a 100 mL graduated cylinder, expressed as g/cm^3^ [28]. 

The OD colour intensity (CI) analysis was performed using the CIELAB colour system (lightness L*; redness a*; and yellowness b*) with an X-Rite 962/964 handheld spectrophotometer supported by an illuminant head D65 (X-Rite, Inc., Grand Rapids, MI, USA). An 8 mL OD sample was transferred into a cuvette, and the obtained colour values were used in the whiteness index (WI) calculation as described previously by Jeske et al. [13]. The WI was calculated from the means of three measurements for each colour parameter.

The Brix degree (°Bx) was measured in the ODs after they were diluted with distilled water five times and centrifuged at 2295× *g* for 10 min. This test assesses the total soluble solids content (TSSC), which includes proteins, lipids, glucides, mineral salts, vitamins, organic acids, pigments, and other substances in ODs [29,30]. The °Bx readings were taken using an Abbe refractometer (DR-A1, Atago, Bellevue, DC, USA) at 20 °C.

### 2.6. Sensory Evaluation

The sensory differential evaluation was conducted using the degree of difference test (DOD) as described by Costell [31]. In this evaluation, the selected ODnp and ODap samples were compared against the ODc sample for specific attributes such as colour, sweetness, mouthfeel, consistency, and overall difference. Prior to the evaluation, the ODs were stored at 4 °C for 24 h. The assessment involved 17 trained panellists (7 males and 10 females), ranging in age from 20 to 53 years. Informed consent was obtained from all the assessors involved in the study before their sensory evaluation process. The samples were anonymised with 3-digit codes and evaluated on a 5-point just-about-right (JAR) scale using Fizz acquisition software version 2.51C (Biosystems, Couternon, Bourgogne, France).

### 2.7. Statistical Analyses

The experiments were carried out in triplicate, and the data were presented as mean ± standard deviation (SD, *n* = 3). The analytical results were processed using Microsoft Excel^®^ 2021. Statistical analyses were performed with OriginPro 2021b (OriginLab Corp., Northampton, MA, USA). A one-way analysis of variance (ANOVA) and Tukey’s post hoc tests were used to determine differences in the nutritional composition and physicochemical–rheological parameters of the ODc and the OD treated with either neutral or acidic protease over different intervals, at a significance level of α = 0.05. Principal component analysis (PCA) for the OD’s nutritional composition and yield, along with the Pearson correlation matrix for the OD’s rheological parameters and nutritional composition, were generated using XLSTAT 2023 software (XLSTAT statistical and data analysis solution, New York, NY, USA).

The sensory evaluation results were analysed using an ANOVA with a post hoc least significant difference (LSD) test, followed by a descriptive graphical representation of the means and SDs of the attributes for each type of OD, utilising Fizz sensory software version 2.51C (Biosystems, Couternon, Bourgogne, France).

## 3. Results

### 3.1. Fluctuation in Nutritional Composition within Protease Treatment Intervals

**Figure 2:**
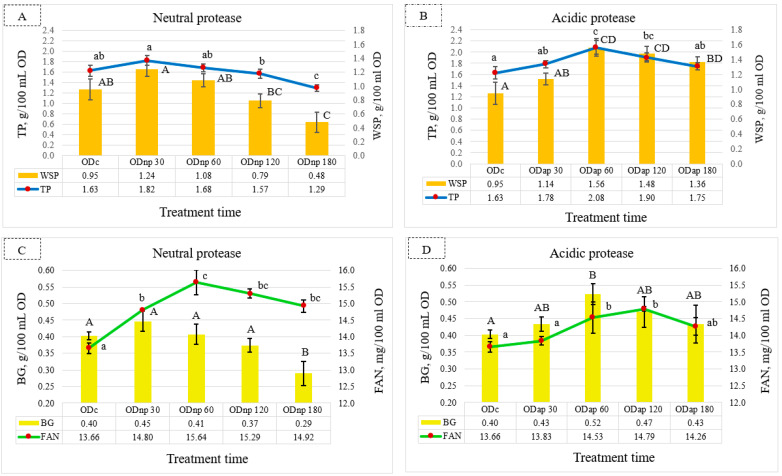
Fluctuation in the nutritional composition of oat drink (OD) based on the duration of protease treatment: changes in total protein (TP) and water-soluble protein (WSP) are shown under neutral protease (NP) treatment—(**A**), and under acidic protease (AP) treatment—(**B**); β-glucan (BG) and free amino nitrogen (FAN) are shown under NP treatment—(**C**), and under AP treatment—(**D**). These parameters were analysed in ODc (control OD), and ODnp/ap 30, 60, 120, and 180 are ODs treated for 30, 60, 120, and 180 min with neutral (NP) or acidic (AP) proteases, respectively. In each graph, distinct letters indicate significant differences at the 5% level (*p* ≤ 0.05) determined by Tukey’s test, where lowercase letters denote TP and FAN and uppercase letters denote WSP and BG. All results are expressed as mean ± SD bars (*n* = 3).

Compared to the ODc sample, the NP treatment results (Figure 2A) showed the maximum increments in TP and WSP contents by 0.19 and 0.29 g/100 mL OD, respectively, after 30 min of hydrolysis. However, this slight increase in TP and WSP contents was not statistically significant (*p* > 0.05). As also shown in Figure 2A, extended treatment with NP for 180 min resulted in a significant decrease in the TP and WSP contents of OD. However, the AP treatment showed a significant effect (*p* < 0.05) for maximising the TP and WSP contents after 60 min of hydrolysis, where their increments were 0.45 and 0.61 g/100 mL OD, respectively, compared to the ODc sample (Figure 2B). After the AP treatment for 180 min, the TP and WSP contents were still higher than their contents in the ODc, but the TP value was not significantly different (*p* > 0.05).

Meanwhile, the BG content in the ODs changed during each protease treatment interval. The NP treatment for 30 min showed a slight increase in BG content by 0.05 g/100 mL OD, which was not significantly different (*p* > 0.05) compared to the ODc sample (Figure 2C). In contrast, the results of the ODnp180 indicate a significant (*p* < 0.05) decline in BG content. Figure 2D illustrates that the AP treatment for 60 min had a significant effect (*p* < 0.05) on increasing the BG content by 0.12 g/100 mL OD compared to the ODc sample. However, extended AP hydrolysis resulted in a slight decrease in BG content after 180 min but was not significantly different compared to the ODc sample.

Nonetheless, FAN content in the OD reached its peak with NP and AP treatment after 60 and 120 min, respectively (Figure 2C,D). Figure 2C,D reveal that the NP treatment for 60 min exhibited a more pronounced effect than the AP treatment for 120 min in terms of increasing the FAN content of ODs by 0.85 mg/100 mL OD, whereas both proteases significantly (*p* < 0.05) increased the FAN content. 

According to these findings, the optimal duration for OD processing using NP and AP is 30 and 60 min, respectively, in order to maximise most of the nutritional components.

### 3.2. Effect of Protease Application on the Yield and Total Solids of Oat Drink

**Figure 3:**
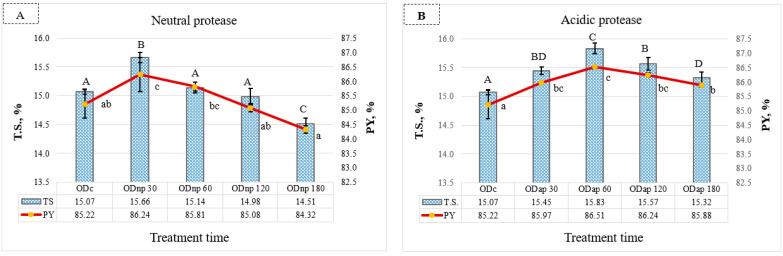
Changes in the oat drinks’ (ODs) production yield (PY) and their total solid content (T.S.) during processing with neutral protease (NP)—(**A**) and acidic protease (AP)—(**B**): ODc represents the control OD, and ODnp/ap 30, 60, 120, and 180 are ODs treated for 30, 60, 120, and 180 min with neutral (NP) or acidic (AP) proteases, respectively. In both graphs, distinct letters indicate significant differences at the 5% level (*p* ≤ 0.05) determined by Tukey’s test, where lowercase letters denote PY and uppercase letters denote T.S. All results are expressed as mean ± SD bars (*n* = 3).

The NP treatment for 30 min significantly increased the T.S. content by 0.59% [*w*/*v*] in OD (*p* ≤ 0.05) and also raised the PY (1.02% [*v*/*v*]) compared to the ODc (Figure 3A). The effects of the AP treatment are demonstrated in Figure 3B, where enzymatic hydrolysis for 60 min had the most significant impact (*p* < 0.05) on enhancing both the T.S. content and the PY of OD by 0.76% and 1.29%, respectively, compared to the ODc. Extended protease treatment up to 180 min resulted in a decrease in both T.S. and PY (Figure 3A,B). NP treatment resulted in a more pronounced drop in T.S. and PY, which were significantly lower than those of the ODc.

**Figure 4:**
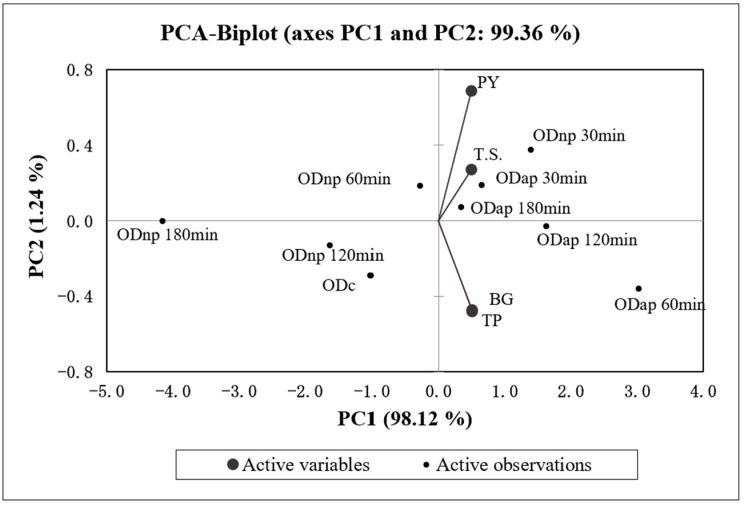
The biplot of principal component analysis (PCA) for the four variables (TP—total protein, BG—β-glucan, T.S.—total solids content, and PY—production yield) of the control oat drink (ODc) and oat drinks treated with neutral protease (ODnp) and acidic protease (ODap) at different intervals (30, 60, 120, and 180 min). PC1 and PC2 account for 98.12% and 1.24% of the total variance, respectively.

Figure 4 presents the correlation analysis and the PCA, which indicate a statistically significant (*p* < 0.05) and strong positive correlation (r > 0.96) between all four variables (TP, BG, T.S., and PY) of OD. The PCA specifically demonstrates a strong positive correlation (r = 0.998) between the changes in TP and BG contents in OD during hydrolysis by both proteases. Additionally, the PCA results (Figure 4) demonstrate that the increase in TP content in ODs during the protease treatment is positively correlated with the T.S. content (r = 0.976) and the PY (r = 0.963) of OD products. 

According to the first principal component axis (PC1), ODnp30 and all AP-treated samples are grouped on the positive side, indicating higher values for the variables compared to the control sample. Conversely, ODnp60, 120, and 180 samples are grouped on the negative side along with the control sample, reflecting similar or lower values for the variables. 

### 3.3. Effect of Protease Treatments on Oat Drink’s Physicochemical–Rheological Properties

**Figure 5:**
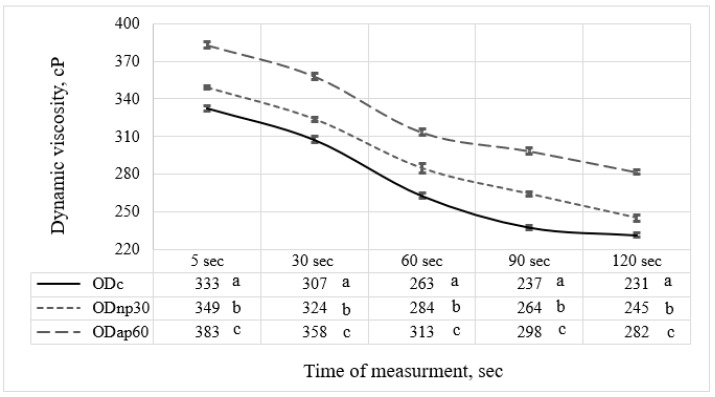
The viscosity of different oat drink (OD) types—ODc (control OD), ODnp30 (OD treated with neutral protease for 30 min), and ODap60 (OD treated with acidic protease for 60 min)—was measured using a rheometer over 120 s. Data in the same column with different letters are significantly different (*p* ≤ 0.05) according to Tukey’s test. The standard deviation of the viscosity results is represented by the error bars.

The selected oat drinks, ODnp30 and ODap60, based on their nutritional composition, were evaluated for changes in rheological and physicochemical parameters resulting from their altered chemical composition. The results were then compared with those of the ODc. The viscosity results recorded after 120 s of measurement for both ODs treated with proteases showed that the AP treatment had a notable effect on elevating the OD viscosity by 1.22-fold, while the NP treatment resulted in only a 1.06-fold increase compared to the ODc (Figure 5). Both protease treatments showed significant differences (*p* < 0.05) in modifying the viscosity of ODs throughout the measurement period compared to ODc. The time-dependent viscosity decrease at a constant shear rate (Figure 5) suggests that ODs exhibit shear-thinning behaviour, as previously reported by Deswal et al. [30].

**Figure 6:**
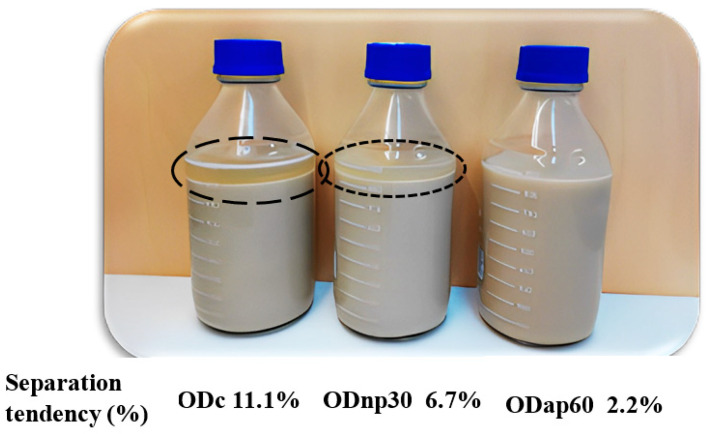
Visual stability of oat drink (OD) samples stored at 4 °C for 17 days without shaking. The current results represent the following OD types: ODc—control OD; ODnp30—OD treated with neutral protease (NP) for 30 min; and ODap60—OD treated with acidic protease (AP) for 60 min. The separation phase of the ODs is marked by different circles based on the potential degree of separation (high-potential—long dash; low-potential—square dot) and their separation tendencies are indicated in the image.

The improvement in the viscosity of ODs as a result of protease treatment impacted the product stability properties, as presented in Figure 6 and Table 1. A visual assessment of the ODs’ storage stability at 4 °C for 17 days without shaking revealed changes in the product’s stability during this storage period. Specifically, phase separation was evident in the ODc, less pronounced in the ODnp30, and almost absent in the ODap60, with separation tendencies of 11.1%, 6.7%, and 2.2%, respectively (Figure 6). 

Consequently, the reduction in the separation rate (Table 1) also indicated enhanced product stability. The AP treatment for 60 min reduced the OD’s separation rate by 17.11%/h, whereas the NP treatment for 30 min reduced it only by 5.37%/h, compared to the ODc, demonstrating statistically significant differences (*p* < 0.05) in both cases. Changes in product viscosity due to each protease treatment significantly influenced product density. ODc had the lowest density, whereas ODnp30 and ODap60 exhibited higher densities, with ODap60 showing a significant (*p* < 0.05) increase in density by 0.025 g/cm^3^.

In parallel to improvements in product stability, the observed changes in the chemical composition of the ODs during hydrolysis impacted significantly (*p* < 0.05) the Brix degree and the whiteness index (WI) of the products. The Brix degree, indicating the total soluble solids content, showed the highest increase of 0.87 °Bx in the ODs following 60 min of AP hydrolysis (Table 1). Conversely, the products’ WI decreased as a result of the enriched nutritional content. Both enzymatic treatments resulted in a significant (*p* < 0.05) decline in the WI of ODs, with decreases of 2.54 and 4.38 units for the NP and AP treatments, respectively.

The Pearson’s correlation coefficient test (Table 2) demonstrates that increasing the BG content of ODs using proteases significantly (*p* < 0.05) raised the viscosity (r = 0.921) and reduced the Sep. rate (r = −0.947) of the products. However, the darker colour of the ODs, as estimated by the WI, and the increment in TSSC, expressed by °Bx, significantly correlated (*p* < 0.05) with changes in WSP content, i.e., whiteness index (r = −0.915) and Brix degree (r = 0.836). The OD product’s WI and Sep. rate are negatively correlated with all other measured parameters of the same product. Correlation analysis (Table 2) shows that changes in the OD’s viscosity are positively correlated to BG and WSP contents, whereas the Sep. rate is negatively correlated to BG and WSP. The viscosity and Sep. rate were more strongly correlated to BG content than to WSP. However, the variation in OD’s WI is more dependent on its WSP content than its BG content.

The correlation analysis (Table 2) indicates that the enhancements in OD viscosity correlate strongly positively (r = 0.853) with its density and negatively (r = −0.975) with its Sep. rate. Additionally, the colour intensity presented as WI of ODs is significantly (*p* < 0.05) more influenced by changes in their Brix degree = TSSC (r = −0.936) than by changes in their WSP content (r = −0.915). These observations have potential industrial value in helping manufacturers optimise the product according to its primary purpose and consumer demand.

### 3.4. Differential Sensory Assessment of Oat Drinks

The analysis of sensory evaluation results using Fizz software highlighted a significant difference (*p* < 0.05) in sensory criteria between the ODc and the OD treated with AP. In contrast, the sensory criteria of the OD treated with NP were not significantly different (*p* = 0.642) from the control sample. The statistical analysis revealed that the variance in the ODnp results stemmed from different opinions among assessors, while in the case of ODap, the variance resulted from the distinctly different attributes of the product.

**Figure 7:**
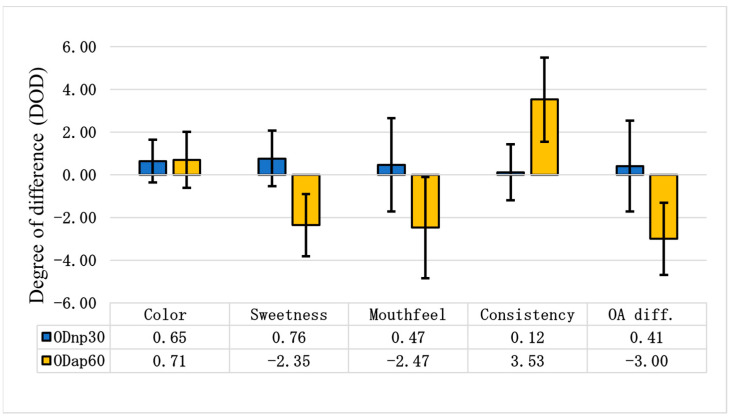
Differential sensory tests of oat drinks (ODs). The differential sensory test was evaluated using the degree of difference (DOD) to assess changes in each attribute between ODnp30 (OD treated with neutral protease for 30 min) or ODap60 (OD treated with acidic protease for 60 min) and the control sample (ODc). A DOD of 0.00 represents the ODc, with positive values indicating a better attribute and negative values indicating a worse attribute. The samples were compared based on the following attributes: colour, sweetness, mouthfeel, consistency, and overall difference (OA diff.). The graphical representation shows the mean results of 17 assessors ± SD.

Figure 7 illustrates the degree of difference in various sensory attributes for ODnp30 and ODap60 compared to ODc. When compared to the control, evaluators noted that ODnp30 became slightly sweeter and less oaty, with a slightly viscous mouthfeel. However, ODap60 was perceived as tasteless and non-sweet, with a strong, ropy, and gluey mouthfeel. According to the overall (OA) difference degree, the NP treatment slightly improved the sensory attributes of the OD, increasing consumer acceptance of the product; meanwhile, the AP treatment clearly worsened the sensory attributes of the OD, leading to a decrease in its consumer acceptance (Figure 7).

## 4. Discussion

The profile of TP and WSP content changes in ODs indicated different hydrolysis rates for each protease examined. Neutral protease exhibited a fast and potent hydrolysis rate for oat proteins due to the optimum pH environment of the OD, resulting in maximum TP and WSP contents after 30 min of treatment. However, these content enhancements declined continuously with extended hydrolysis time. Similar observations were previously highlighted in the partial hydrolysis of oat protein by a controlled trypsin hydrolysis duration (30 min) to enhance protein solubility and gelation properties [8]. Shahi et al. [32] noted that the degree of protein hydrolysis using alcalase and pancreatin increases with the processing time of each protease, resulting in shorter peptide chains that aggregate and precipitate more easily. In the case of AP, the OD’s pH limited enzyme activity, achieving maximum TP and WSP increments after 60 min of treatment. Kumar et al. [33] demonstrated the pH-dependent alteration in acid protease stability and activity, where its activity declines with the pH increase while remaining stable up to pH 6.5. These results emphasise the efficiency of protease incorporation in OD processing for partially hydrolysing the oat protein, enhancing its solubility, and augmenting its extraction yield in the final products. Guan et al. [34] and Nieto-Nieto et al. [8] explained that oat protein-limited hydrolysis is a preferable tool for modifying the secondary and tertiary structures of oat protein, improving functionality and solubility; however, excessive enzymatic hydrolysis can impair protein solubility. The results of the optimum treatment with each protease enzyme demonstrated a higher increment in the WSP than in the TP by 0.1 g/100 mL OD with NP and by 0.16 g/100 mL OD with AP, proving the ability of proteases to convert existing non-soluble proteins into soluble proteins [15,35]. 

As a consequence of oat protein hydrolysis, the OD’s free amino nitrogen (FAN) content increased. However, the changes in FAN content exhibited a different pattern than the changes in TP and WSP. The increment in the degree of hydrolysis with processing time was explained by Shahi et al. [32], which led the FAN content in the ODs to reach its peak after 60 min with NP treatment and after 120 min with AP treatment. The fluctuation in TP content in the OD product showed a strong positive correlation with its BG content, where the BG content alteration pattern during NP and AP hydrolysis mirrored the changes seen in TP and WSP. This finding is consistent with Patra et al. [9], who confirmed that OD products containing a high amount of intact β-glucan tend to have a high protein concentration. This strong correlation results from the firm covalent bond between oat proteins and β-glucan, forming a complex structure known as the ‘protein-β-glucan linkage’ [17,18]. The variation in the chemical composition of the ODs synergistically impacted both production yield and total solids. In this study, the utilisation of proteases in oat drink production was a key factor in improving production yield during the filtration process and the T.S. content of the final product within 30 and 60 min of NP and AP treatment, respectively.

The enhancement of WSP and BG contents in the selected ODnp30 and ODap60 showed a strong positive correlation with their viscosity. Thus, the apparent viscosity changes appear to result from the preservation of intact β-glucan in the OD [9,15] and the partial hydrolysis of its proteins, which modified their functionality and strengthened their water-binding capacity [7,35,36]. The graphical representation, as also described by Deswal et al. [30], of the OD’s viscosity illustrates its non-Newtonian pseudoplastic rheological behaviour (shear-thinning viscosity), attributed to the colloidal nature of the OD and the breakdown/alignment of its constituent colloid and polymer chains (e.g., fat, starch, protein). This flow behaviour complements the observations of Patra et al. [9], which justify the presence of intact β-glucan in OD, which results in a thick product with assertive shear-thinning behaviour. In contrast, enzymatic hydrolysis of β-glucan changed the behaviour of the ODs to near-Newtonian flow. Person’s correlation analysis proved that the β-glucan increment in the OD had a more substantial effect on enhancing its viscosity than the WSP increment.

Viscosity improvement had a potent effect on the physical stability parameters of the ODs, which was reflected in cold storage stability and separation rate. The highest viscosity of ODap60 prevented any phase separation in the OD product during its 17-day shelf life under refrigerated storage. The phenomenon of separation in oat drink products has previously been described by Jeske et al. [13], who explained that oat-based drink systems are polydisperse and thus prone to instability. The formulation of commercial drinks usually contains hydrocolloids such as various gum thickeners and stabilisers, e.g., carrageenan or xanthan gum, to increase the viscosity and thus discourage separation [3,13,37]. The OD product with intact β-glucan processed from bran, studied by Patra et al. [9], was highly viscous and stable throughout the storage time at 20 °C for 14 days. The presence of intact oat β-glucan in oat drinks could potentially prevent inherent separation. The separation phenomena of the oat-based drink system have been explained by the thermodynamic incompatibilities that force separation [38]. Thus, less viscous oat drinks (such as ODc) exhibit a distinct separation phase, indicated by a high separation tendency (11.1%), compared to more viscous products, particularly ODap60, which is a stable drink with a low separation tendency (2.2%).

Patent US 2015/0351432A1 [16] confirmed the effect of highly extracted proteins and water-soluble protein contents in oat drinks on improving emulsion stability and reducing sedimentation rates. The same observation was noticed in this study, where the separation rate in ODap60 was reduced by 17.11%/h in parallel with the increment in its WSP and TP contents compared to ODc. Therefore, incorporating protease enzymes in the manufacture of oat beverages is a promising tool for achieving consumer-predictable product viscosity and stability, consistent with Chen’s [15] findings. An increase in viscosity in OD products has a stronger impact on reducing product separation rate than increasing product density. Both the BG and WSP contents of OD had nearly the same potency correlation with the product’s density. The density-based separation mechanism of OD is commonly explained by the Stokes–Einstein equation, which states that separation velocity is affected by particle diameter, the densities of the dispersed and continuous phases, gravity, and the viscosity of the continuous phase [26,37].

The increase in WSP content in ODs was associated with a reduction in the product’s whiteness index (WI), intensifying their brownish colour. The distinctive brown colour of the oat drink is a result of natural pigments or Maillard reaction products formed during heat treatment, which also affects consumer acceptability [3,5,39,40]. The main natural pigments in oats that can impact the drink’s colour are anthocyanins and lutein [40,41,42], but further research is required to identify the primary natural pigments influencing the colour of oat drinks. The incorporation of proteases in oat beverage production supported the release of natural brown pigments in the products, likely bound to oat protein. This observation complements the findings of Brückner-Gühmann et al. [43] on enzymatic hydrolysis to modify the gelling properties of oat proteins by alcalase and trypsin under alkaline conditions, resulting in an intensely brownish protein hydrolysate and its gel. The Brix degrees of the ODs, reflecting their TSS changes, were equally affected by the increase in BG and WSP content in the same product. The protease treatment in oat drink processing can be considered a tool for boosting the release of oat-soluble ingredients, including soluble fibres (β-glucan, arabinan, and xylan), free sugars, amino nitrogens, vitamins, polyphenols, and other ingredients that enrich the nutritional value of OD products.

Consumer acceptance is the primary factor driving product perspectives. The differential sensorial assessment showed that neutral protease treatment produced a slightly sweeter and less oaty product with a slightly viscous mouthfeel, making it highly suitable as a drinkable oat beverage product. In contrast, acidic protease treatment produced a tasteless, highly viscous product with a gluey or ropy mouthfeel, unsuitable for the OD beverage experience due to the high increase in intact β-glucan and WSP in the OD product. Patra et al. [9] explained that the presence of highly intact β-glucan in ODs results in a highly viscous, gel-like product unsuitable for thin beverage applications. The high concentration of oatmeal (16%) in the initial slurry, higher than in commercial OD products, must be considered. Conversely, OD treated with acidic protease might serve as the foundation for non-dairy yoghurt or fermented oat-based products and other viscous plant-based products, such as puddings and smoothies. Further studies are required to deepen the understanding of the structural and techno-functional changes that occur in ODs during treatment with different proteases or protein deamidases.

## 5. Conclusions

The nutritional composition of ODs is a crucial factor in the physicochemical and sensory features of the product. Protease treatment is considered a promising tool for improving the compositional parameters of ODs, especially their water-soluble proteins and β-glucan. The optimal treatment duration for OD processing with neutral and acidic proteases was 30 min and 60 min, respectively, for maximising the TP content and its solubility, which was strongly positively correlated with increasing β-glucan content in the same product. A highly nutritious OD product with total protein (2.08 g/100 mL OD), water-soluble protein (1.56 g/100 mL OD), and β-glucan (0.52 g/100 mL OD) contents was produced by the AP treatment for 60 min, achieving a maximum production yield of 86.51%. Compared to the ODc, the AP treatment for 60 min had the highest effect on elevating the OD’s viscosity by 1.22-fold, the density by 0.025 g/cm^3^, and the Brix (TSS content) by 0.87 °Bx. Additionally, the AP treatment decreased the OD’s separation rate by 17.11%/h, boosted its storage stability for 17 days, and prevented the appearance of phase separation. However, the pronounced increment in the WSP and BG contents of OD treated with AP had an adverse effect on its sensory characteristics, resulting in a gluey, highly viscous, and unsuitable drinkable product. Meanwhile, the OD treated with NP demonstrated higher consumer acceptance, with a slight enhancement in the sensorial and nutritional profiles of the final ODs. The industrial processing of OD with controlled protease treatment requires accuracy to balance the increase in its nutritional composition with its physicochemical and sensory attributes to avoid a loss of consumer acceptance and product marketability.

## Figures and Tables

**Table 1:** Changes in rheological and physicochemical parameters of oat drinks (ODs) during neutral protease (NP) and acidic protease (AP) treatments.

OD Types	Separation Rate (%/h)	Density (g/cm^3^)	Brix (°Bx)	Whiteness Index
ODc	19.24 ± 1.827 ^a^	1.048 ± 0.005 ^a^	14.30 ± 0.125 ^a^	52.79 ± 0.044 ^a^
ODnp30	13.87 ± 2.054 ^b^	1.055 ± 0.004 ^ab^	14.62 ± 0.071 ^b^	50.25 ± 0.135 ^b^
ODap60	2.13 ± 1.120 ^c^	1.073 ± 0.012 ^b^	15.17 ± 0.165 ^c^	48.41 ± 0.069 ^c^

The results represent the mean of each parameter in ODs: ODc—control OD (light grey row), ODnp30 (OD treated with neutral protease for 30 min), and ODap60 (OD treated with acidic protease for 60 min), and their standard deviation values (±SD, *n* = 3). Data in the same column with different letters are significantly different (*p* ≤ 0.05) according to Tukey’s test.

**Table 2:** Pearson’s correlation coefficients between eight variables of oat drinks (OD): total protein (TP), water-soluble protein (WSP), β-glucan (BG), viscosity, density, separation rate (Sep. rate), whiteness index (WI), and Brix degree.

	TP	WSP	BG	Viscosity	Density	Sep. rate	WI	Brix
**TP**	1	*<0.001*	*<0.001*	*0.001*	*0.009*	*0.001*	*0.001*	*0.009*
**WSP**	0.932	1	*<0.001*	*0.001*	*0.005*	*<0.001*	*0.001*	*0.005*
**BG**	0.970	0.924	1	*<0.001*	*0.007*	*<0.001*	*0.001*	*0.006*
**Viscosity**	0.892	0.891	0.921	1	*0.003*	*<0.001*	*<0.001*	*<0.001*
**Density**	0.804	0.835	0.821	0.853	1	*0.002*	*0.005*	*0.028*
**Sep. rate**	−0.901	−0.927	−0.947	−0.975	−0.869	1	*<0.001*	*0.001*
**WI**	−0.887	−0.915	−0.886	−0.934	−0.833	0.934	1	*<0.001*
**Brix**	0.801	0.836	0.829	0.924	0.723	−0.913	−0.936	1

The lower left side represents the correlation (r), with white-coloured boxes indicating a positive correlation and grey-coloured boxes indicating a negative correlation. The upper right side of the table shows the significance of these correlations, where values in italics indicate statistically significant differences (*p* < 0.05).

## Data Availability

The original contributions presented in the study are included in the article; further inquiries can be directed to the corresponding author.
